# Threshold-modifying effect of the systemic inflammatory response index on kidney function decline in hypertensive patients

**DOI:** 10.1186/s40001-024-01804-9

**Published:** 2024-03-27

**Authors:** Xing Wei, Jing Wei, Jun Feng, Chao Li, Zhipeng Zhang, Ben Hu, Nv Long, Chunmiao Luo

**Affiliations:** 1 Department of Cardiology, The Second People’s Hospital of Hefei, Hefei Hospital Affiliated to Anhui Medical University, Hefei, 230011 Anhui China; 2 The Fifth Clinical School of Medicine, Anhui Medical University, Hefei, 230032 Anhui China

**Keywords:** Hypertension, NHANES, Systemic inflammation response index, eGFR, ACR

## Abstract

**Background:**

Chronic kidney disease (decreased kidney function) is common in hypertensive patients. The SIRI is a novel immune biomarker. We investigated the correlation between the SIRI and kidney function in hypertensive patients.

**Methods:**

The present study analyzed data from participants who suffered from hypertension in the NHANES from 2009 to 2018. Multivariate regression analysis and subgroup analysis were used to clarify whether the SIRI was an independent risk factor for decreased kidney function. RCSs were utilized to evaluate the correlation between the SIRI and the eGFR and between the SIRI and the ACR. In addition, we modeled the mediating effect of the SIRI on the eGFR and the ACR using blood pressure as a mediating variable.

**Results:**

The highest SIRI was an independent risk factor for a decreased eGFR [odds ratio (OR) = 1.46, 95% CI (1.15, 1.86)] and an increased ACR [OR = 2.26, 95% CI (1.82, 2.82)] when the lowest quartile was used as the reference. The RCS results indicated an inverted U-shaped relationship between the SIRI and the eGFR and between the SIRI and the ACR (the inflection points were 1.86 and 3.09, respectively). The mediation effect analysis revealed that the SIRI was the main factor influencing kidney function, and diastolic blood pressure was a mediating variable. In particular, there was a fully mediating effect between the SIRI and UCr, with a mediating effect value of -0.61 (-0.90, -0.36).

**Conclusions:**

The association between the SIRI and renal function in hypertensive patients was significant and was particularly dominated by the association between the SIRI and the ACR. This difference may be due to the mediating effect of diastolic blood pressure.

**Supplementary Information:**

The online version contains supplementary material available at 10.1186/s40001-024-01804-9.

## Introduction

Hypertension is a significant determinant of kidney disease progression and is exacerbated by kidney failure [1]. In addition, both hypertension and chronic kidney disease (CKD) are independent risk factors for cardiovascular disease (CVD), and the morbidity and mortality of CVD significantly increase when both coexist [2, 3]. Multiple mechanisms contribute to the development of hypertensive nephropathy; as the estimated glomerular filtration rate (eGFR) decreases, the renin–angiotensin–aldosterone system is upregulated, promoting sodium retention and increasing blood pressure, exacerbating hypertensive kidney damage [2, 4]. In addition, several factors, including oxidative stress and the resulting relative renal hypoxia, may further exacerbate the development of hypertension and CKD [5]. CKD is characterized by reduced renal function, defined as an eGFR of less than 60 ml/min/1.73 m^2^ and/or a urinary albumin (UAlb)-to-creatinine ratio (ACR) of more than 30 mg/g for more than three months [6, 7]. A decreased eGFR reflects the extent of kidney damage [8], and increased UAlb excretion is an independent predictor of the progression of kidney damage [9]. Proteinuria quantification can also stratify the risk of kidney damage and can be utilized as a marker of response to treatment [10]. Several studies have shown that the ACR is superior to 24-h UAlb excretion for predicting CKD [11]. CKD affects many adults worldwide, and a persistent chronic low-grade inflammatory state is a significant contributor to its development and progression [12]. The immune-inflammatory mechanism is now recognized as a component of the multifactorial etiology of hypertension and related organ damage [13]. Chronic low-grade inflammation leads to elevated blood pressure in various experimental models of hypertension, resulting in target organ damage [14]. The systemic inflammatory response index (SIRI) has been proposed as a novel inflammatory biomarker involving single inflammatory markers such as neutrophils, monocytes, and lymphocytes that can more accurately predict poor prognosis in patients with GI tumors [15, 16]. Previous studies have shown that induction of neutrophil apoptosis by CD39 on regulatory T cells during RAAS activation attenuates hypertension, suggesting that neutrophils may be one of the factors contributing to elevated blood pressure [17]. Furthermore, monocytes can release pro-inflammatory cytokines, such as IL-6, IL-1β, and TNF-α [18]. All of the above inflammatory response mechanisms can play a role in promoting the development of hypertension. Chronic low-grade inflammation has been associated with a variety of health outcomes, and recent investigations have shown that SIRI is related to the development of all-cause mortality and cardiovascular mortality in adults [19]. However, there are no studies on the association between the SIRI and renal impairment in hypertensive patients, and this study aimed to investigate the association between the SIRI and kidney function in adult hypertensive patients in the U.S. using data from the National Health and Nutrition Examination Survey (NHANES).

## Materials and methods

### Research population

**Fig. 1:**
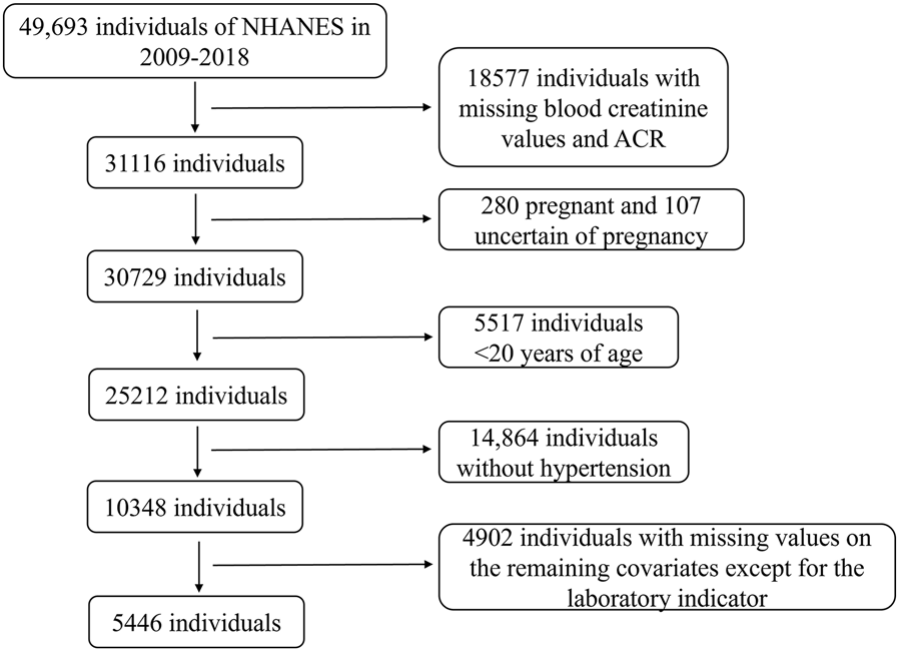
Research flow chart

We investigated a total of 49,693 participants in five cycles of the NHANES from 2009 through 2018. Among these participants, those who met the following criteria were excluded from the analysis: individuals with missing creatinine (Cr) and ACR data; pregnant individuals and female individuals who could not specify whether they were pregnant or not pregnant; adolescent individuals aged less than 20 years old; nonhypertensive individuals; and individuals with missing data on race, marital status, education level, BMI, and personal history. The final sample consisted of 5446 individuals (Fig. 1).

### Statement

NHANES is a nationally representative survey of the general population conducted by the National Center for Health Statistics (NCHS). Detailed data is obtainable at the https://www.cdc.gov/nchs/nhanes/. All study protocols of NHANES were approved by the Research Ethics Review Board of the NCHS. The study was exempt from ethical review because the NHANES database is open to the public. All participants provided written informed consent. The study adheres to the Declaration of Helsinki.

### Exposure variables and outcome variables

Routine blood samples from the study participants were analyzed via automated hematology analysis equipment. The exposure variable (the SIRI) was calculated as (neutrophil count) × (monocyte count)/(lymphocyte count) [15].

The primary outcome of this study was an eGFR ≤ 60 mL/minute/1.73 m^2^ [The glomerular filtration rate was estimated using the following formula [20]: 175 × Scr^−1.154^ × age^−0.203^ × 1.212 (if black) × 0.742 (if female)]. The secondary outcome was defined as an ACR ≥ 30 mg/g. Serum Cr concentrations were analyzed by the Jaffe rate method with a Beckman UniCel DxC800 Synchron (Beckman, Fullerton, CA, USA). UAlb was measured from a spot sample using a solid-phase fluorescent immunoassay. Urinary creatinine (UCr) was measured using Roche/Hitachi Modular P Chemistry and Roche/Hitachi Cobas 6000 chemistry Analyzer using an enzymatic (creatinase) method immunoassay.

### Covariates and definitions

Baseline demographic information about the participants in this study was obtained from demographic data. History of alcohol consumption, smoking, diabetes, and hypertension were obtained from the participants’ questionnaires. Regarding laboratory indices that may be potential confounders, platelet (PLT), hemoglobin (Hb), alanine aminotransferase (ALT), aspartate aminotransferase (AST), high-density lipoprotein cholesterol (HDL-C), total cholesterol (TC), triglycerides (TG), albumin (ALB), glucose, and glycosylated hemoglobin (HbA1c) were collected in this study.

Hypertension was defined as 1. having been explicitly told that they had hypertension previously, 2. being on antihypertensive medication, 3. we calculated the mean of systolic and diastolic blood pressure (DBP) using the patient’s three resting state measurements for participants who did not have a history of this condition or who were taking medication for this condition, and we chose to use the mean of the participant’s blood pressure from the first two resting state measurements for participants who had a third missing measurement, and were defined as having high blood pressure when the participants were defined as hypertensive when the mean systolic blood pressure (SBP) was ≥ 140 mmHg or the mean DBP was ≥ 90 mmHg. Definition of diabetes mellitus: 1. fasting blood glucose ≥ 11.1 mmol/L, 2. HbA1c ≥ 6.5%, 3. previous notification of diabetes mellitus, and 4. ongoing insulin use. Smoking history was defined as “Never” for participants who had not smoked more than 100 cigarettes in their lifetime, "Smoking former" for those who had smoked more than 100 cigarettes in their lifetime but were no longer current smokers, and “Smoking now” for those who were current smokers. Drinking history was defined as “Rarely drinker” for participants who had not consumed 12 drinks in their lifetime, “Light drinker” for participants who had consumed more than 12 drinks in their lifetime but not more than 12 drinks in a year, and “Excessive drinker” for participants who had consumed more than 12 drinks in a year.

### Grouping

This investigation was divided into two groups based on the primary outcome event (eGFR ≤ 60 mL/minute/1.73 m^2^ and eGFR > 60 mL/minute/1.73 m^2^). It was divided into two groups based on secondary outcome (ACR < 30 mg/g and ACR ≥ 30 mg/g). Participants were categorized into four grades based on SIRI level quartiles: Q1 (*n* = 1361, SIRI ≤ 0.73), Q2 (*n* = 1362, 0.73 < SIRI ≤ 1.11), Q3 (*n* = 1361, 1.11 < SIRI ≤ 1.66), and Q4 (*n* = 1362, SIRI > 1.66).

### Statistical analysis

In this research, all continuous numerical variables are expressed as medians (quartiles) after the normality test and were analyzed using the nonparametric rank-sum test. Categorical variables were compared using the Chi-square test. Binary logistic regression analyses were performed with group Q1 as the reference group. In the regression model with eGFR ≤ 60 mL/minute/1.73 m^2^ as the endpoint event, the minimum-adjusted model was adjusted for age, sex, smoking status, drinking status, history of diabetes, race, education, and marital status. Fully adjusted models were adjusted for age, sex, smoking status, drinking status, history of diabetes mellitus, race, education, marriage, BMI, HDL-C, ALT, AST, TG, TC, ALB, PLT, Hb, glucose, and HbA1c. In regression models with ACR ≥ 30 mg/g as the outcome, the minimally adjusted model was adjusted for age, sex, smoking history, drinking history, history of diabetes mellitus, race, education level, and marital status, and the fully adjusted model was adjusted for age, sex, smoking history, drinking history, history of diabetes mellitus, race, education level, marital status, BMI, ALT, HDL-C, TG, TC, ALB, PLT, Hb, glucose, and HbA1c. The median of the four sets of SIRI was taken for a dummy variable setting and then tested for trend. We utilized the restricted cubic spline curves (RCS) to fit smoothed curves and perform threshold effects analysis (the adjustment strategy was the same as the fully adjusted regression model). This was followed by interaction tests in subgroup analyses. To further investigate the relationship between SIRI, blood pressure, and renal function, we developed a mediated effect model using SBP and DBP as mediating variables. In addition, we performed sensitivity analyses for two populations (1. Excluding those with recent-onset hypertension. 2. Excluding those with current blood pressure below 140/90 mmHg). This study discarded laboratory indicators with missing values > 20%, and for laboratory indicators with missing values < 20%, this study used multiple sampling interpolation to interpolate the variables. In this study, we used unweighted estimation. Because this study limited the inclusion members to the hypertensive population, after excluding participants who did not meet the inclusion criteria, the inclusion members were unevenly distributed from cycle to cycle, resulting in large variations in the weights of the combined samples. Unweighted estimation is recommended when sample weights vary significantly or when covariates used to calculate weights (such as age, gender, and race) are already included in the regression model [21]. Statistical analyses were performed using R Studio (version R4.2.3) and EmpowerStats (version 4.1). All tests were two-tailed, and *P* < 0.05 was considered to indicate statistical significance.

## Results

### Comparison of participants' baseline information

This study enrolled 5446 participants, including 2721 males (49.96%, mean age 58.22 ± 15.31 years) and 2725 females (50.04%, mean age 60.43 ± 14.39 years). eGFR levels increased by race, education level, and marital status, and were lower in older, female, diabetic, and non-smoking drinkers. ACR levels increased by race, education level, and marital status. ACR levels increased significantly with advancing age, and albuminuria was more common in diabetes, nonsmoking participants, and participants who consumed alcohol. In addition, The SIRI was substantially greater in patients with a low eGFR and albuminuria (Table 1). Compared with those in the low-level group, patients in the high-SIRI group were generally older; had a greater proportion of males; had a greater prevalence of obesity, diabetes, smoking and drinking; were non-Hispanic white; were more likely to have elevated platelet counts and hemoglobin, triglyceride and fasting glucose levels; were more likely to have a greater ACR; and were more likely to have lower high-density lipoprotein cholesterol, total cholesterol and ALB levels as well as a lower eGFR (Additional file 1: Table S1).

**Table 1:** Comparison of participants' baseline information

Features	eGFR > 60 mL/minute/1.73 m^2^ (*N* = 4490)	eGFR ≤ 60 mL/minute/1.73 m^2^ (*N* = 956)	*P* value	ACR < 30 mg/g (*N* = 4328)	ACR ≥ 30 mg/g (*N* = 1118)	*P* value
Age (years)	59.00 (47.00, 68.00)	72.00 (63.00, 80.00)	< 0.001	60.00 (48.00, 70.00)	66.00 (55.00, 76.00)	< 0.001
Gender (%)			0.001			0.301
Men	2288 (50.96%)	433 (45.29%)		2147 (49.61%)	574 (51.34%)	
Women	2202 (49.04%)	523 (54.71%)		2181 (50.39%)	544 (48.66%)	
BMI (kg/m^2^)	29.60 (25.90, 34.60)	29.30 (25.70, 34.00)	0.200	29.50 (25.90, 34.40)	30.10 (25.80, 35.30)	0.124
Diabetes (%)			< 0.001			< 0.001
No	3359 (74.81%)	566 (59.21%)		3342 (77.22%)	583 (52.15%)	
Yes	1131 (25.19%)	390 (40.79%)		986 (22.78%)	535 (47.85%)	
Smoking status (%)			< 0.001			0.093
Never	2312 (51.49%)	467 (48.85%)		2241 (51.78%)	538 (48.12%)	
Smoking former	1269 (28.26%)	338 (35.36%)		1257 (29.04%)	350 (31.31%)	
Smoking now	909 (20.24%)	151 (15.79%)		830 (19.18%)	230 (20.57%)	
Drinking status (%)			< 0.001			0.046
Rarely drinker	714 (15.90%)	190 (19.87%)		708 (16.36%)	196 (17.53%)	
Light drinker	621 (13.83%)	164 (17.15%)		602 (13.91%)	183 (16.37%)	
Excessive drinker	3155 (70.27%)	602 (62.97%)		3018 (69.73%)	739 (66.10%)	
Race/Ethnicity (%)			< 0.001			0.013
Mexican American	567 (12.63%)	71 (7.43%)		482 (11.14%)	156 (13.95%)	
Other Hispanic	498 (11.09%)	56 (5.86%)		446 (10.30%)	108 (9.66%)	
Non-Hispanic White	1708 (38.04%)	405 (42.36%)		1717 (39.67%)	396 (35.42%)	
Non-Hispanic Black	1121 (24.97%)	341 (35.67%)		1140 (26.34%)	322 (28.80%)	
Other Race	596 (13.27%)	83 (8.68%)		543 (12.55%)	136 (12.16%)	
Education (%)			< 0.001			< 0.001
Less than high school	1120 (24.94%)	297 (31.07%)		1044 (24.12%)	373 (33.36%)	
High school	1069 (23.81%)	225 (23.54%)		1029 (23.78%)	265 (23.70%)	
More than high school	2301 (51.25%)	434 (45.40%)		2255 (52.10%)	480 (42.93%)	
Marital status (%)			< 0.001			< 0.001
Never married	563 (12.54%)	87 (9.10%)		540 (12.48%)	110 (9.84%)	
Widowed/divorced/separated	1284 (28.60%)	412 (43.10%)		1271 (29.37%)	425 (38.01%)	
Married/livingwith partner	2643 (58.86%)	457 (47.80%)		2517 (58.16%)	583 (52.15%)	
WBC(× 10^9^/L)	7.00 (5.80, 8.50)	7.00 (5.80, 8.40)	0.475	6.90 (5.70, 8.40)	7.30 (6.10, 8.80)	< 0.001
Neutrophil (× 10^9^/L)	4.00 (3.20, 5.20)	4.10 (3.30, 5.20)	0.540	4.00 (3.10, 5.10)	4.40 (3.40, 5.60)	< 0.001
Lymphocyte(× 10^9^/L)	2.00 (1.60, 2.50)	1.90 (1.50, 2.40)	< 0.001	2.00 (1.60, 2.50)	1.90 (1.50, 2.50)	< 0.001
Monocyte (× 10^9^/L)	0.60 (0.40, 0.70)	0.60 (0.50, 0.70)	0.003	0.55 (0.40, 0.70)	0.60 (0.50, 0.70)	< 0.001
PLT(× 10^9^/L)	230.00 (195.00, 271.00)	215.00 (179.00, 257.00)	< 0.001	228.00 (193.00, 270.00)	221.00 (186.00, 268.00)	0.004
Hb(g/dL)	14.00 (13.10, 15.00)	13.10 (12.00, 14.10)	< 0.001	13.90 (12.90, 15.00)	13.60 (12.40, 14.67)	< 0.001
ALT (IU/L)	22.00 (17.00, 29.00)	18.00 (14.00, 24.00)	< 0.001	21.00 (17.00, 29.00)	20.00 (15.00, 27.00)	< 0.001
AST (IU/L)	24.00 (20.00, 29.00)	23.00 (20.00, 27.00)	0.004	23.00 (20.00, 28.00)	23.00 (19.00, 29.00)	0.338
HDL-C (mmol/L)	1.29 (1.06, 1.58)	1.27 (1.03, 1.58)	0.247	1.29 (1.09, 1.58)	1.25 (1.01, 1.55)	< 0.001
TG (mmol/L)	1.50 (0.99, 2.30)	1.57 (1.06, 2.29)	0.147	1.49 (0.99, 2.26)	1.61 (1.06, 2.47)	< 0.001
TC (mmol/L)	4.96 (4.21, 5.71)	4.66 (3.90, 5.44)	< 0.001	4.91 (4.19, 5.69)	4.81 (4.03, 5.66)	0.014
ALB (g/L)	43.00 (40.00, 45.00)	41.00 (39.00, 44.00)	< 0.001	42.00 (40.00, 45.00)	42.00 (39.00, 44.00)	< 0.001
Glucose (mmol/L)	5.44 (4.94, 6.33)	5.72 (5.05, 7.05)	< 0.001	5.44 (4.94, 6.22)	5.94 (5.16, 8.26)	< 0.001
HbA1c (%)	5.70 (5.40, 6.20)	6.00 (5.60, 6.70)	< 0.001	5.70 (5.40, 6.10)	6.10 (5.60, 7.20)	< 0.001
SIRI	1.09 (0.73, 1.62)	1.26 (0.81, 1.88)	< 0.001	1.08 (0.72, 1.60)	1.30 (0.85, 1.98)	< 0.001

All continuous numeric variables are expressed using medians (quartiles) and categorical variables are quantified as numbers (percentages)

*PLT* platelets, *ALT* alanine transaminase, *AST* aspartate transaminase, *HDL-C* high-density lipoprotein-cholesterol, *TG* triglycerides, *TC* total cholesterol, *ALB* Albumin, *HbA1c* glycosylated hemoglobin, *SIRI* Systemic Inflammation Response Index

### Correlation between the SIRI and kidney function

Among the two unadjusted univariate regression models with an eGFR ≤ 60 mL/minute/1.73 m^2^ and an ACR ≥ 30 mg/g as the outcome event, the SIRI was associated with a decrease in the eGFR (≤ 60 mL/minute/1.73 m^2^) (Q1 vs. Q2: HR, 0.99 [95% CI 0.80, 1.23]; Q3: HR, 1.18 [95% CI 0.96, 1.45]; Q4: HR, 1.70 [95% CI 1.40, 2.07]; *P* for trend < 0.001), elevated ACR (≥ 30 mg/g) (Q1 vs. Q2: HR, 1.22 [95% CI 1.00, 1.50]; Q3: HR, 1.44 [95% CI 1.18, 1.76]; Q4: HR, 2.29 [95% CI 1.89, 2.76]; *P* for trend < 0.001) were associated. The results of the regression models adjusted for all confounders were consistent, with a high SIRI associated with a decreased eGFR (≤ 60 mL/minute/1.73 m2) (Q1 vs. Q4: HR, 1.46 [95% CI 1.15, 1.86]) and an elevated ACR (≥ 30 mg/g) (Q1 vs. Q4: HR, 2.26 [95% CI 1.82, 2.82]) (Table 2).

**Table 2:** Regression models with eGFR ≤ 60 mL/minute/1.73 m^2^ and ACR ≥ 30 mg/g as outcome events

Features	eGFR (OR (95%CI) *P*)			ACR (OR (95%CI) *P*)		
	Mode 1	Mode 2	Model 3	Mode 1	Mode 2	Model 3
SIRI [median(quartile)]						
Q1[0.543 (≤ 0.73)]	Ref.	Ref.	Ref.	Ref.	Ref.	Ref.
Q2[0.919 (0.73–1.11)]	0.99 (0.80, 1.23)	1.02 (0.81, 1.29)	1.01 (0.79, 1.27)	1.22 (1.00, 1.50)	1.28 (1.04, 1.59)*	1.28 (1.03, 1.59)*
Q3[1.344 (1.11–1.66)]	1.18 (0.96, 1.45)	1.22 (0.97, 1.53)	1.23 (0.97, 1.56)	1.44 (1.18, 1.76)***	1.47 (1.19, 1.82)***	1.48 (1.19, 1.84)***
Q4[2.250 (> 1.66)]	1.70 (1.40, 2.07)***	1.51 (1.20, 1.90)***	1.46 (1.15, 1.86)**	2.29 (1.89, 2.76)***	2.23 (1.80, 2.75)***	2.26 (1.82, 2.82)***
*P* for trend	< 0.001	< 0.001	< 0.001	< 0.001	< 0.001	< 0.001

Multifactor regression model was developed using group Q1 as the reference group. Calculate the median row trend test for each group

*OR* odds ratio, *CI* confidence interval

^*^P < 0.05

^**^P < 0.01

^***^P < 0.001

**Fig. 2:**
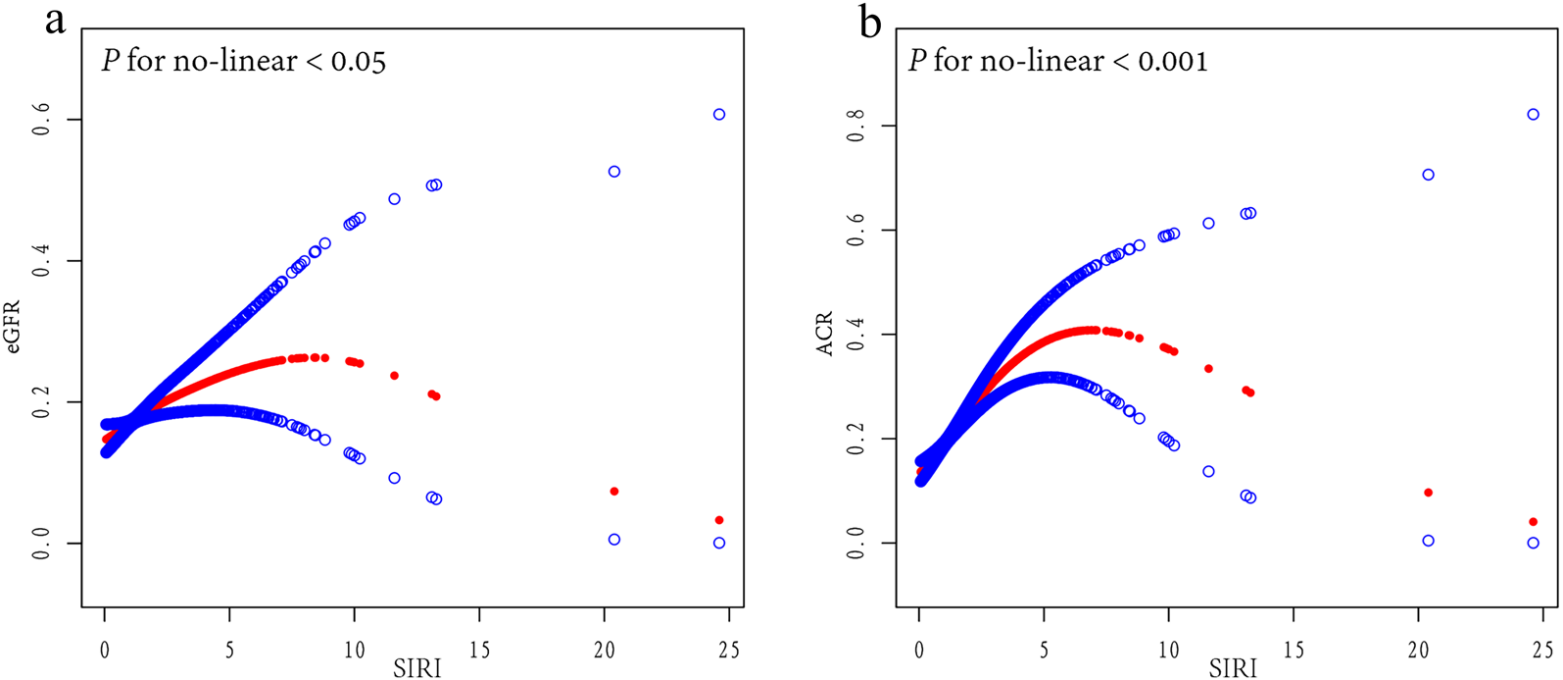
Restricted cubic spline curve. The adjustment strategy is the same as the fully adjusted regression model. **a** Fitting a smoothed curve with SIRI as the independent variable and eGFR as the dependent variable results in an inverted U-shaped curve (inflection point 1.86). **b** Fitting a smoothed curve with SIRI as the independent variable and ACR as the dependent variable results in an inverted U-shaped curve (inflection point 3.09)

The RCS results showed that the SIRI was associated with an inverted U-shaped nonlinear increase in the risk of both a decreased eGFR (*P* = 0.014) and an elevated ACR (*P* < 0.001) (the adjustment strategy was the same as that for the fully adjusted logistic regression model) (Fig. 2). We further performed a threshold effect analysis, which revealed that in the standard linear regression model, SIRI was associated with a decrease in the eGFR (*P* = 0.0451) and an increase in the ACR (*P* < 0.001), respectively; after adjusting for confounders, we built a two-stage linear regression model, which detected an inverted “U” shape with inflection points of 1.86 and 3.09 for the eGFR and the ACR, respectively (Table 3).

**Table 3:** Analysis of threshold effects

	eGFR (mL/minute/1.73 m^2^)	ACR (mg/g)
Standard linear regression model	1.08 (1.00, 1.15) 0.044	1.22(1.14, 1.30) < 0.001
Two-stage regression models		
Inflection point (K)	1.86	3.09
< K	1.37 (1.15, 1.64) < 0.001	1.52(1.37, 1.68) < 0.001
> K	0.99 (0.90, 1.08) 0.771	0.96(0.86, 1.07) 0.445
log-likelihood ratio test	0.003	< 0.001

The adjustment strategy is the same as the fully adjusted regression model

### Subgroup analysis

In subgroup analyses, the SIRI was significantly associated with a decreased eGFR in patients with advanced age, female sex, high BMI, diabetes mellitus status, nonsmoking status, and consumption of alcohol, with interactions showing that the results were robust (Additional file 1: Table S2). In addition, the SIRI was significantly associated with an elevated ACR in individuals who were older, male, had a high BMI, were married, had diabetes mellitus, and consumed alcohol. According to the analyses of the other subgroups excluding alcohol consumption, the interaction results remained robust (Additional file 1: Table S3).

### Mediation effect analysis with blood pressure as a mediating variable

**Fig. 3:**
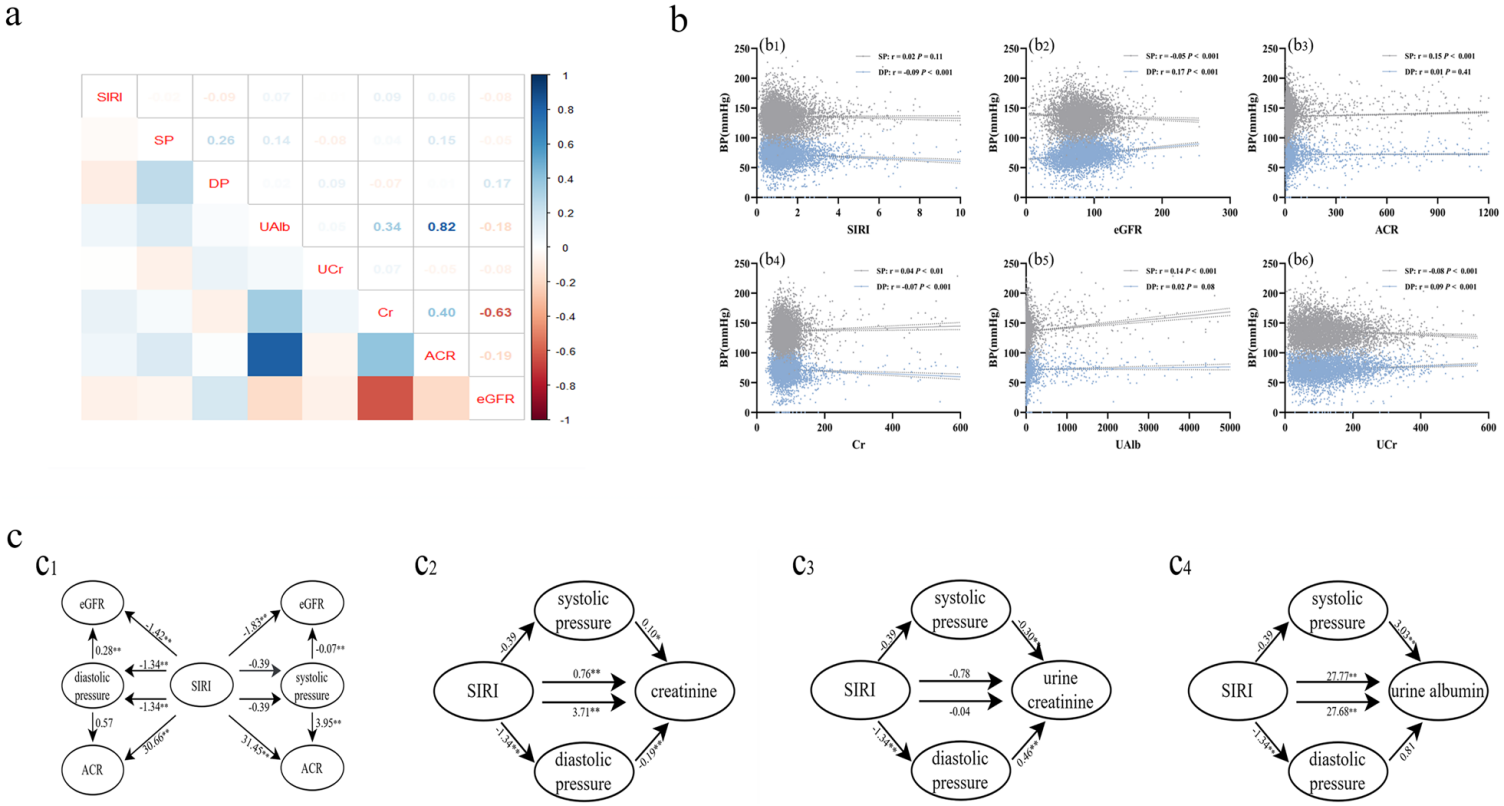
Analysis of intermediation effects**.** r: correlation coefficient, *: *P* < 0.05, **: *P* < 0.01, ***: *P* < 0.001。*SIRI* Systemic Inflammation Response Index, *SBP* systolic blood pressure, *DBP* diastolic blood pressure, *UAlb* urine albumin, *UCr* urinary creatinine, *Cr* creatinine, *ACR* Urine Albumin Creatinine Ratio, *eGFR* estimated glomerular filtration rate. **a** Correlation analysis between SIRI, SBP, DBP and kidney function indicators; **b** Scatter plot of correlation between SBP and DBP as dependent variables and SIRI, eGFR, ACR, Cr, UAlb, UCr; **c** All mediated effects models had SIRI as the independent variable and systolic and diastolic blood pressure as the mediating variables. Model c1 had eGFR and ACR as dependent variables, model c2 had Cr as dependent variable, model c3 had UCr as dependent variable, and model c4 had UAlb as dependent variable

Correlation analysis revealed that the SIRI, SBP, and DBP were weakly correlated with UAlb, UCr, the ACR, and the eGFR (All correlation coefficients *r* < 0.2) (Fig. 3a). Among those correlations, SBP had weak positive correlations with the ACR (*r* = 0.15, *P* < 0.001), Cr (*r* = 0.04, *P* < 0.01), and UAlb (*r* = 0.14, *P* < 0.001) and negative correlations with the eGFR (*r* = − 0.05, *P* < 0.001) and UCr (r = -0.08, *P* < 0.001); DBP was negatively correlated with the eGFR (*r* = 0.17, *P* < 0.001) and UCr (*r* = 0.09, *P* < 0.001) and had weak positive correlations with the SIRI (*r* = − 0.09, *P* < 0.001) and Cr (*r* = − 0.07, *P* < 0.001) (Fig. 3b).

Based on the results, we conducted a mediation effect analysis with the SIRI as the independent variable; SBP and DBP as the mediating variables; and the eGFR, the ACR, Cr, UCr, and UAlb as the dependent variables in this study. The results indicated that a direct effect was observed between the SIRI and the eGFR (*β* = − 1.83, *P* < 0.01) and between the SIRI and the ACR (*β* = 31.45, *P* < 0.01) in the model with SBP as the mediating variable. In the model with DBP as the mediating variable, there was a partial mediating effect between the SIRI and the eGFR, with a mediating effect value of − 0.38 (− 0.53, − 0.25), which accounted for 0.21 of the total impact; however, there was a direct effect model between the SIRI and the ACR (*β* = 30.66, *P* < 0.01). We further tested the mediating effects with Cr, UCr, and UAlb selected as the dependent variables. The results indicated that DBP partially mediated the effect between the SIRI and Cr, and its mediating effect was 0.25 (0.11, 0.43), accounting for 0.06 of the total effect ratio; in addition, DBP and fully mediated the effect between the SIRI and UCr, with a mediating effect of − 0.61 (− 0.90, − 0.36). The rest of the models showed all direct effects and no mediating effects (Fig. 3c).

### Sensitivity analysis

Sensitivity analyses were performed in this investigation.1. We excluded people with new-onset hypertension. The results showed that the risk of a decreased eGFR and the risk of an increased ACR associated with the SIRI were greater in the fully adjusted model (eGFR: Q1vs. Q4 (OR, 1.37 [95% CI 1.06, 1.75])), (ACR: Q1vs. Q4 (OR, 1.37 [95% CI 1.06, 1.75])) (Additional file 1: Table S4). 2. We performed regression analyses again for those with mean blood pressure higher than 140/90 mmHg in this research. The results showed that the SIRI was more strongly correlated with a decrease in the eGFR according to the fully adjusted regression model (Q1vs. Q4 (OR, 1.81 [95% CI 1.25, 2.62]) (Additional file 1: Table S5). 3. We performed propensity score matching based on confounders between the SIRI and the outcomes. As shown in Additional file 1: Tables S6 and S7, the results remain solid, and the difference in SIRI between the two groups is statistically significant (*P* < 0.001).

## Discussion

In the present study, we demonstrated that a high SIRI was strongly associated with kidney insufficiency in hypertensive patients. The correlation coefficient remained significant after adjusting for confounding factors such as baseline population information and laboratory data. We further fitted the smoothed curves and found that the SIRI had inverted U-shaped relationships with a decreased eGFR and an increased ACR in hypertensive patients (inflection points of 1.86 and 3.09, respectively). In the subgroup analyses, we applied an identical adjustment strategy, and the results were robust. In addition, to further analyze the association between the SIRI, blood pressure, and kidney function, we performed a mediation effect analysis. The results revealed that in the model with DBP as the mediating variable, DBP had a partial mediating effect on the relationship between the SIRI and the eGFR. In addition, DBP had a partial mediating effect on the relationship between the SIRI and Cr. Moreover, DBP fully mediated the relationship between the SIRI and UCr. This finding implies that the SIRI may modulate kidney function through DBP in hypertensive patients. However, we did not observe the same effect on SBP. This deserves further study. The pathophysiology of hypertensive kidney injury is complex and results from a variety of factors. Chronic low-grade inflammation, reduced number of kidney units, expansion of extracellular fluid volume due to increased water and sodium retention, overactivity of the sympathetic and RASS systems, and endothelial dysfunction have been suggested as potential mechanisms [12, 22, 23]. Meanwhile, kidney injury, loss of functional kidney units, and increased RASS activity greatly increase the salt sensitivity of blood pressure, which can contribute to further elevation of blood pressure, thus creating a vicious cycle. These changes within the microcirculation eventually result in chronic ischemia and fibrosis and are characterized by increased proteinuria and blood creatinine [24].

Many diseases, such as metabolic syndrome, cardiovascular disease, and diabetes mellitus, are associated with chronic low-grade inflammation [25]. Distinct inflammatory markers each play different roles in the kidney. TNF-α activates the endothelial inflammatory response, leading to capillary leakage and allowing entry of immune cells. Simultaneously, immune cells (monocytes and macrophages) are attracted to MCP-1. During this time, neutrophils are chemotactically attracted to IL-8, and IL-23 upregulates the proliferation of Th17 cells, which triggers a more proinflammatory response [26]. The SIRI integrates neutrophils, monocytes, and lymphocytes, reflecting the interaction between immunity and inflammation [15, 19]. In a retrospective cohort study from China on the SIRI and long-term outcomes of patients with type B aortic dissection, when the researchers adjusted for confounders, they identified a significant correlation between the SIRI and poor prognosis [27]. One study demonstrated that the SIRI can be used as a predictor of stroke risk in elderly hypertensive patients [28]. In addition, in a recent retrospective cohort study, researchers used the MIMIC-III database to analyze the predictive value of SIRI in the prognosis of patients with stroke, and the study showed that, after adjusting for several covariates, SIRI was correlated with all-cause mortality of patients with stroke, and as the SIRI rises, the mortality rate also increases [29]. This finding demonstrated that the SIRI is related not only to the occurrence of stroke but also to the prognosis of stroke. The above studies suggest that the SIRI is closely related to cardiovascular and cerebrovascular diseases. Two recent retrospective analyses of diabetic patients suggest that the SIRI is an independent risk factor for deteriorating kidney function [30, 31]. In earlier studies, increased neutrophil and monocyte counts as well as decreased lymphocyte counts were associated with decreased kidney function. This may clarify the relationship between the SIRI and kidney function [32–34]. In addition, in a prospective clinical study exploring postoperative metastasis of kidney cell carcinoma, researchers found that the SIRI had high predictive value for metastasis (AUC of 0.737) [35]. However, there are no studies on the correlation between the SIRI and renal insufficiency in hypertensive patients.

Under pathological conditions, increased production of reactive oxygen species (ROS) triggers oxidative stress, which plays a role in vascular changes associated with hypertension, including endothelial dysfunction, vascular reactivity, and arterial remodeling [36]. Furthermore, oxidative stress is associated with inflammation, as ROS production activates inflammatory cells and enhances the production of inflammatory mediators. Relatively, inflammation increases ROS release, creating a vicious cycle [37]. ROS has an undertaking relationship between blood pressure and inflammation. And there is an intrinsic correlation between blood pressure and kidney function. Above-target SBP or no nocturnal drop is associated with a higher risk of cardiovascular and kidney disease progression in patients with CKD, regardless of ambulatory blood pressure, a multicenter cohort study suggests [38]. The high prevalence of hypertension at all stages of CKD and the dual benefit of effective antihypertensive therapy in reducing renal and cardiovascular risk amply demonstrate the bidirectional relationship between hypertension and renal function^1^.

An intrinsic link exists between inflammation, blood pressure, and kidney function. We employed mediation effect analysis to clarify the relationship between the SIRI, blood pressure, and renal function. In the mediation effect analysis, it was observed that DBP mediated the relationship between the SIRI and kidney function, playing a fully mediating role in the association between the SIRI and UCr. These findings indicate that changes in DBP play a more critical role in the decline in renal function in hypertensive patients. In the present study, we noticed a more significant correlation between the SIRI and the ACR than between the SIRI and the eGFR. An elevated ACR is a marker of early kidney disease [10]. The above analysis revealed that early in the disease course, special attention should be given to the SIRI in hypertensive patients.

This research has several limitations. First, we used only the mean of three blood pressure measurements for each of the participants as the study variable in the mediation effect analysis, but to accurately assess the mediating effect of participants’ blood pressure, ambulatory blood pressure values should have been used as the study variable. Second, the participants included in the analysis of this study were all hypertensive patients, and some of them may have taken relevant medications for antihypertensive purposes, which is a confounding factor, but we had no way of knowing the application of appropriate medications by the participants. Third, the blood cell-based assay was performed only once, and the concentration of these blood cells may be affected by other factors and changes. We believe that further relevant cohort studies are necessary to explore the associations of the SIRI with blood pressure and kidney function in hypertensive patients.

## Conclusion

The present investigation confirmed a correlation between the SIRI and kidney function in hypertensive patients. This difference may be mediated by increases in diastolic blood pressure leading to decreased kidney function. These findings indicate that immune inflammation should be treated in hypertensive patients.

### Supplementary Information

**Additional file 1: Table S1. **Baseline information table based on SIRI quartiles. **Table S2. **Subgroup analysis of outcome events with eGFR ≤60 mL/minute/1.73 m^2^. **Table S3. **Subgroup analysis of outcome events with ACR≥30 mg/g. **Table S4. **Regression analysis of exclusion of people with new-onset hypertension. **Table S5.** Regression analysis with exclusion of people with blood pressure less than 140/90 mmHg. **Table S6.** Propensity score matching for outcome events with eGFR ≤60 mL/minute/1.73 m^2^. **Table S7.** Propensity score matching for outcome events with ACR≥30 mg/g.

## Data Availability

This study analyzed datasets from the publicly available database NHANES from 2009 to 2018. These data can be found here: https://www.cdc.gov/nchs/nhanes/.

## Abbreviations

SIRI: Systemic inflammatory response index
NHANES: National Health and Nutrition Examination Survey
NCHS: National Center for Health Statistics
eGFR: Estimated glomerular filtration rate
ACR: Albumin creatinine ratio
UAlb: Urinary albumin
UCr: Urinary creatinine
CKD: Chronic kidney disease
CVD: Cardiovascular disease
Cr: Creatinine
PLT: Platelet
Hb: Hemoglobin
ALT: Alanine aminotransferase
AST: Aspartate aminotransferase
HDL-C: High-density lipoprotein cholesterol
TC: Total cholesterol
TG: Triglycerides
ALB: Albumin
HbA1c: Glycosylated hemoglobin
DBP: Diastolic blood pressure
SBP: Systolic blood pressure
RCS: Restricted cubic spline curves
OR: Odds ratio
CI: Confidence interval
