# Supplementary material for: Threshold-modifying effect of the systemic inflammatory response index on kidney function decline in hypertensive patients
Source: Eur J Med Res. 2024 Mar 27;29:202. doi: 10.1186/s40001-024-01804-9 (PMC10967104; doi:10.1186/s40001-024-01804-9)
Supplement: Supplementary file 1 — Additional file 1: Table S1. Baseline information table based on SIRI quartiles. Table S2. Subgroup analysis of outcome events with eGFR ≤60 mL/minute/1.73 m2. Table S3. Subgroup analysis of outcome events with ACR≥30 mg/g. Table S4. Regression analysis of exclusion of people with new-onset hypertension. Table S5. Regression analysis with exclusion of people with blood pressure less than 140/90 mmHg. Table S6. Propensity score matching for outcome events with eGFR ≤60 mL/minute/1.73 m2. Table S7. Propensity score matching for outcome events with ACR≥30 mg/g. [file 40001_2024_1804_MOESM1_ESM.docx]

**Table S1** Baseline information table based on SIRI quartiles

| Features | | SIRI (Quartiles) | |  | |
| --- | --- | --- | --- | --- | --- |
|  | Q1(≤0.73) (1361) | Q2(0.73-1.11) (1362) | Q3(1.11-1.66) (1361) | Q4(>1.66) (1362) | *P*-value |
| Age (years) | 59.00(48.00, 67.00) | 61.00(50.00, 70.00) | 61.00(48.00, 71.00) | 64.00(52.00, 76.00) | <0.001 |
| Gender (%) |  |  |  |  | <0.001 |
| Men | 573 (42.10%) | 617 (45.30%) | 705 (51.80%) | 826 (60.65%) |  |
| Women | 788 (57.90%) | 745 (54.70%) | 656 (48.20%) | 536 (39.35%) |  |
| BMI (kg/m^2^) | 29.30(25.60, 33.80) | 29.40(25.90, 34.40) | 30.20(26.40, 35.00) | 29.60(25.70, 34.98) | 0.003 |
| Diabetes (%) |  |  |  |  | <0.001 |
| No | 1012 (74.36%) | 1016 (74.60%) | 968 (71.12%) | 929 (68.21%) |  |
| Yes | 349 (25.64%) | 346 (25.40%) | 393 (28.88%) | 433 (31.79%) |  |
| Smoking status (%) |  |  |  |  | <0.001 |
| Never | 769 (56.50%) | 739 (54.26%) | 693 (50.92%) | 578 (42.44%) |  |
| Smoking former | 345 (25.35%) | 397 (29.15%) | 393 (28.88%) | 472 (34.65%) |  |
| Smoking now | 247 (18.15%) | 226 (16.59%) | 275 (20.21%) | 312 (22.91%) |  |
| Drinking status (%) |  |  |  |  | <0.001 |
| Rarely drinker | 244 (17.93%) | 243 (17.84%) | 231 (16.97%) | 186 (13.66%) |  |
| Light drinker | 233 (17.12%) | 195 (14.32%) | 184 (13.52%) | 173 (12.70%) |  |
| Excessive drinker | 884 (64.95%) | 924 (67.84%) | 946 (69.51%) | 1003 (73.64%) |  |
| Race/Ethnicity (%) |  |  |  |  | <0.001 |
| Mexican American | 129 (9.48%) | 165 (12.11%) | 191 (14.03%) | 153 (11.23%) |  |
| Other Hispanic | 119 (8.74%) | 161 (11.82%) | 138 (10.14%) | 136 (9.99%) |  |
| Non-Hispanic White | 282 (20.72%) | 495 (36.34%) | 588 (43.20%) | 748 (54.92%) |  |
| Non-Hispanic Black | 624 (45.85%) | 354 (25.99%) | 288 (21.16%) | 196 (14.39%) |  |
| Other Race | 207 (15.21%) | 187 (13.73%) | 156 (11.46%) | 129 (9.47%) |  |
| Education (%) |  |  |  |  | 0.729 |
| Less than high school | 364 (26.75%) | 342 (25.11%) | 354 (26.01%) | 357 (26.21%) |  |
| High school | 322 (23.66%) | 313 (22.98%) | 318 (23.37%) | 341 (25.04%) |  |
| More than high school | 675 (49.60%) | 707 (51.91%) | 689 (50.62%) | 664 (48.75%) |  |
| Marital status (%) |  |  |  |  | 0.004 |
| Never married | 191 (14.03%) | 158 (11.60%) | 151 (11.09%) | 150 (11.01%) |  |
| Widowed/Divorced/  Separated | 413 (30.35%) | 414 (30.40%) | 396 (29.10%) | 473 (34.73%) |  |
| Married/Living  with partner | 757 (55.62%) | 790 (58.00%) | 814 (59.81%) | 739 (54.26%) |  |
| PLT(×10^9^/L) | 221.00 (187.00-259.00) | 226.00 (192.00-265.00) | 230.00 (195.00-271.00) | 234.00 (194.00-280.75) | <0.001 |
| Hb (g/L) | 13.60 (12.70-14.70) | 13.90 (12.90-14.70) | 14.00 (13.00-15.00) | 14.10 (12.90-15.10) | <0.001 |
| ALT (IU/L) | 21.00 (16.00-28.00) | 21.00 (17.00-29.00) | 21.00 (16.00-29.00) | 21.00 (16.00-28.00) | 0.314 |
| AST (IU/L) | 23.00 (20.00-28.00) | 24.00 (20.00-29.00) | 23.00 (20.00-28.00) | 23.00 (20.00-28.00) | 0.401 |
| HDL-C (mmol/L) | 1.34 (1.11-1.66) | 1.32 (1.09-1.60) | 1.24 (1.03-1.53) | 1.24 (1.01-1.53) | <0.001 |
| TG (mmol/L) | 1.34 (0.90-2.04) | 1.52 (1.04-2.40) | 1.59 (1.05-2.34) | 1.59 (1.06-2.39) | <0.001 |
| TC (mmol/L) | 4.99 (4.32-5.74) | 4.99 (4.27-5.71) | 4.89 (4.09-5.69) | 4.71 (3.96-5.48) | <0.001 |
| ALB (g/L) | 43.00 (41.00-45.00) | 42.00 (40.00-44.00) | 42.00 (40.00-44.00) | 42.00 (40.00-44.00) | <0.001 |
| Glucose (mmol/L) | 5.44 (5.00-6.27) | 5.44 (4.94-6.32) | 5.61 (5.00-6.55) | 5.55 (5.00-6.83) | <0.001 |
| HbA1c (%) | 5.80 (5.40-6.20) | 5.75 (5.40-6.10) | 5.80 (5.50-6.30) | 5.80 (5.40-6.30) | 0.094 |
| Cr(umol/L) | 76.91 (64.53-91.94) | 77.79 (65.42-92.82) | 78.68 (66.30-95.47) | 83.10 (69.84-100.78) | <0.001 |
| UAlb(mg/L) | 9.20 (4.70-20.60) | 8.90 (4.50-23.03) | 11.20 (5.10-25.80) | 14.20 (6.30-38.00) | <0.001 |
| UCr(mg/dL) | 107.00 (63.00-164.00) | 97.00 (55.00-154.75) | 106.00 (64.00-160.00) | 102.00 (62.00-156.00) | 0.002 |
| ACR (mg/g) | 8.55 (5.35-17.89) | 9.22 (5.46-20.31) | 9.58 (5.89-22.61) | 12.78 (6.71-35.74) | <0.001 |
| eGFR (mL/minute/1.73 m^2^) | 82.15 (67.63-97.43) | 81.81 (67.00-98.12) | 81.54 (65.79-98.44) | 79.15 (61.80-95.49) | <0.001 |

All continuous numeric variables are expressed using medians (quartiles) and categorical variables are quantified as numbers (percentages).

PLT: platelets, ALT: alanine transaminase, AST: aspartate transaminase, HDL-C: high-density lipoprotein-cholesterol, TG: triglycerides, TC: total cholesterol, ALB: Albumin, HbA1c: glycosylated hemoglobin, Cr: creatinine, UAlb: urine albumin, UCr: urinary creatinine.

**Table S2** Subgroup analysis of outcome events with eGFR ≤60 mL/minute/1.73 m^2^

| **Freatures** | **N** | **Q1** | **Q2** | **Q3** | **Q4** | ***P* for interaction** |
| --- | --- | --- | --- | --- | --- | --- |
| Age |  |  |  |  |  | 0.9126 |
| ≤60 | 2612 | Ref | 1.05 (0.67, 1.63) | 1.15 (0.74, 1.79) | 1.62 (1.02, 2.58) |  |
| ＞60 | 2834 | Ref | 1.06 (0.80, 1.39) | 1.39 (1.05, 1.83) | 1.67 (1.27, 2.21) |  |
| Gender |  |  |  |  |  | 0.3157 |
| Men | 2721 | Ref | 0.85 (0.58, 1.25) | 1.28 (0.89, 1.85) | 1.33 (0.92, 1.92) |  |
| Women | 2725 | Ref | 1.14 (0.84, 1.54) | 1.16 (0.85, 1.60) | 1.64 (1.18, 2.29) |  |
| BMI |  |  |  |  |  | 0.8448 |
| ≤25 | 1102 | Ref | 1.19 (0.70, 2.00) | 1.01 (0.58, 1.76) | 1.42 (0.82, 2.43) |  |
| 25-30 | 1757 | Ref | 0.91 (0.60, 1.39) | 1.24 (0.81, 1.88) | 1.44 (0.94, 2.20) |  |
| ＞30 | 2587 | Ref | 0.96 (0.67, 1.36) | 1.33 (0.94, 1.87) | 1.51 (1.05, 2.17) |  |
| Marital status (%) |  |  |  |  |  | 0.1124 |
| Never married | 650 | Ref | 1.80 (0.86, 3.77) | 1.94 (0.87, 4.31) | 1.77 (0.79, 3.97) |  |
| Widowed/Divorced/ | 1696 | Ref | 1.11 (0.76, 1.63) | 1.15 (0.78, 1.69) | 1.90 (1.30, 2.78) |  |
| Separated | 3100 | Ref | 0.84 (0.60, 1.18) | 1.12 (0.80, 1.55) | 1.06 (0.74, 1.51) |  |
| Race/Ethnicity (%) |  |  |  |  |  | 0.4397 |
| Mexican American | 638 | Ref | 0.96 (0.39, 2.39) | 1.57 (0.65, 3.76) | 1.69 (0.66, 4.34) |  |
| Other Hispanic | 554 | Ref | 1.85 (0.54, 6.38) | 2.88 (0.84, 9.85) | 2.77 (0.82, 9.31) |  |
| Non-Hispanic White | 2113 | Ref | 1.08 (0.69, 1.71) | 0.99 (0.63, 1.56) | 1.32 (0.85, 2.03) |  |
| Non-Hispanic Black | 1462 | Ref | 0.87 (0.61, 1.24) | 1.10 (0.76, 1.59) | 1.38 (0.91, 2.09) |  |
| Other Race | 679 | Ref | 1.08 (0.44, 2.66) | 2.97 (1.27, 6.92) | 2.64 (1.08, 6.46) |  |
| Diabetes (%) |  |  |  |  |  | 0.3538 |
| No | 3925 | Ref | 0.91 (0.68, 1.22) | 1.06 (0.79, 1.42) | 1.28 (0.95, 1.73) |  |
| Yes | 1521 | Ref | 1.16 (0.76, 1.76) | 1.69 (1.12, 2.55) | 1.77 (1.16, 2.69) |  |
| Smoking status (%) |  |  |  |  |  | 0.9929 |
| Never | 2779 | Ref | 1.00 (0.72, 1.39) | 1.19 (0.85, 1.67) | 1.50 (1.06, 2.13) |  |
| Smoking former | 1607 | Ref | 1.05 (0.68, 1.62) | 1.28 (0.83, 1.96) | 1.53 (1.00, 2.34) |  |
| Smoking now | 1060 | Ref | 0.90 (0.49, 1.65) | 1.40 (0.78, 2.51) | 1.44 (0.78, 2.67) |  |
| Drinking status (%) |  |  |  |  |  | 0.9313 |
| Rarely drinker | 904 | Ref | 1.11 (0.64, 1.95) | 1.19 (0.67, 2.09) | 1.69 (0.93, 3.08) |  |
| Light drinker | 785 | Ref | 0.94 (0.53, 1.67) | 1.49 (0.85, 2.62) | 1.64 (0.89, 3.01) |  |
| Excessive drinker | 3757 | Ref | 1.00 (0.74, 1.35) | 1.17 (0.86, 1.57) | 1.37 (1.02, 1.86) |  |

The adjustment strategy is the same as the fully adjusted regression model

**Table S3** Subgroup analysis of outcome events with ACR≥30 mg/g

|  | **N** | **Q1** | **Q2** | **Q3** | **Q4** | ***P* for interaction** | |
| --- | --- | --- | --- | --- | --- | --- | --- |
| Age |  |  |  |  |  | 0.8781 |  |
| ≤60 | 2612 | Ref | 1.18 (0.84, 1.64) | 1.41 (1.01, 1.97) | 2.20 (1.56, 3.11) |  |  |
| ＞60 | 2834 | Ref | 1.40 (1.05, 1.87) | 1.62 (1.22, 2.17) | 2.47 (1.86, 3.29) |  |  |
| Gender |  |  |  |  |  | 0.1058 |  |
| Men | 2721 | Ref | 1.60 (1.13, 2.26) | 1.78 (1.27, 2.50) | 3.11 (2.23, 4.33) |  |  |
| Women | 2725 | Ref | 1.11 (0.83, 1.47) | 1.36 (1.02, 1.81) | 1.79 (1.32, 2.42) |  |  |
| BMI |  |  |  |  |  | 0.8285 |  |
| ≤25 | 1102 | Ref | 1.19 (0.74, 1.91) | 1.31 (0.80, 2.16) | 2.32 (1.42, 3.79) |  |  |
| 25-30 | 1757 | Ref | 1.07 (0.73, 1.58) | 1.23 (0.83, 1.82) | 1.93 (1.31, 2.84) |  |  |
| ＞30 | 2587 | Ref | 1.51 (1.09, 2.08) | 1.74 (1.27, 2.39) | 2.55 (1.85, 3.53) |  |  |
| Marital status (%) |  |  |  |  |  | 0.2943 |  |
| Never married | 650 | Ref | 1.30 (0.68, 2.49) | 2.08 (1.09, 3.98) | 1.72 (0.87, 3.39) |  |  |
| Widowed/Divorced/ | 1696 | Ref | 1.06 (0.74, 1.53) | 1.39 (0.97, 2.00) | 1.95 (1.36, 2.80) |  |  |
| Separated | 3100 | Ref | 1.46 (1.07, 1.97) | 1.47 (1.09, 1.99) | 2.67 (1.96, 3.63) |  |  |
| Race/Ethnicity (%) |  |  |  |  |  | 0.1433 |  |
| Mexican American | 638 | Ref | 0.91 (0.48, 1.73) | 1.12 (0.61, 2.09) | 1.77 (0.93, 3.36) |  |  |
| Other Hispanic | 554 | Ref | 0.84 (0.41, 1.72) | 1.73 (0.86, 3.47) | 1.21 (0.59, 2.50) |  |  |
| Non-Hispanic White | 2113 | Ref | 1.86 (1.14, 3.03) | 1.64 (1.01, 2.65) | 2.54 (1.60, 4.03) |  |  |
| Non-Hispanic Black | 1462 | Ref | 1.33 (0.94, 1.88) | 1.71 (1.19, 2.44) | 2.67 (1.80, 3.96) |  |  |
| Other Race | 679 | Ref | 0.95 (0.53, 1.71) | 1.16 (0.63, 2.14) | 2.88 (1.56, 5.33) |  |  |
| Diabetes (%) |  |  |  |  |  | 0.1944 |  |
| No | 3925 | Ref | 1.12 (0.86, 1.47) | 1.22 (0.92, 1.61) | 2.06 (1.56, 2.71) |  |  |
| Yes | 1521 | Ref | 1.60 (1.12, 2.30) | 1.97 (1.38, 2.80) | 2.68 (1.86, 3.85) |  |  |
| Smoking status (%) |  |  |  |  |  | 0.9820 |  |
| Never | 2779 | Ref | 1.33 (0.99, 1.78) | 1.49 (1.10, 2.01) | 2.42 (1.77, 3.30) |  |  |
| Smoking former | 1607 | Ref | 1.43 (0.93, 2.20) | 1.61 (1.05, 2.48) | 2.54 (1.67, 3.87) |  |  |
| Smoking now | 1060 | Ref | 1.16 (0.69, 1.93) | 1.56 (0.96, 2.54) | 2.05 (1.25, 3.36) |  |  |
| Drinking status (%) |  |  |  |  |  | 0.0018 |  |
| Rarely drinker | 904 | Ref | 0.73 (0.45, 1.19) | 0.72 (0.43, 1.18) | 1.04 (0.61, 1.78) |  |  |
| Light drinker | 785 | Ref | 1.95 (1.10, 3.46) | 2.56 (1.45, 4.54) | 5.62 (3.12, 10.14) |  |  |
| Excessive drinker | 3757 | Ref | 1.41 (1.07, 1.85) | 1.62 (1.23, 2.13) | 2.34 (1.78, 3.07) |  |  |

The adjustment strategy is the same as the fully adjusted regression model

**Table S4** Regression analysis of exclusion of people with new-onset hypertension

| **Features** | | **eGFR (OR (95%CI) *P*)** | |  | | **ACR (OR (95%CI) *P)*** | |
| --- | --- | --- | --- | --- | --- | --- | --- |
|  | Mode 1 | Mode 2 | Model 3 |  | Mode 1 | Mode 2 | Model 3 |
| SIRI [median(quartile)] | |  |  |  |  |  |  |
| Q1[0.54 (≤0.74)] | Ref | Ref | Ref |  | Ref | Ref | Ref |
| Q2[0.92 (0.74-1.12)] | 0.95 (0.77, 1.18) | 1.01 (0.80, 1.28) | 0.97 (0.76, 1.24) |  | 1.25 (1.01, 1.54) | 1.32 (1.06, 1.65) * | 1.30 (1.04, 1.63) * |
| Q3[1.34 (1.12-1.66)] | 1.15 (0.93, 1.42) | 1.23 (0.97, 1.56) | 1.19 (0.93, 1.51) |  | 1.51 (1.23, 1.86) *** | 1.56 (1.25, 1.95) *** | 1.54 (1.23, 1.93) *** |
| Q4[2.26 (>1.66)] | 1.62 (1.32, 1.98) *** | 1.49 (1.17, 1.89) ** | 1.37 (1.06, 1.75) * |  | 2.35 (1.93, 2.87) *** | 2.31 (1.86, 2.89) *** | 2.30 (1.83, 2.89) *** |
| *P* for trend | <0.001 | <0.001 | <0.01 |  | <0.001 | <0.001 | <0.001 |

Multifactor regression model was developed using group Q1 as the reference group. Calculate the median row trend test for each group.

OR: odds ratio, CI: confidence interval. ^*^: *P* <0.05, ^**^: *P* <0.01, ^***^: *P* <0.001.

**Table S5** Regression analysis with exclusion of people with blood pressure less than 140/90 mmHg

| **Features** | | **eGFR (OR (95%CI) *P*)** | |  | | **ACR (OR (95%CI) *P)*** | |
| --- | --- | --- | --- | --- | --- | --- | --- |
|  | Mode 1 | Mode 2 | Model 3 |  | Mode 1 | Mode 2 | Model 3 |
| SIRI [median(quartile)] | |  |  |  |  |  |  |
| Q1[0.58 (≤0.74)] | Ref | Ref | Ref |  | Ref | Ref | Ref |
| Q2[0.87 (0.74-1.11)] | 1.22 (0.89, 1.67) | 1.35 (0.95, 1.91) | 1.34 (0.93, 1.92) |  | 1.18 (0.91, 1.55) | 1.21 (0.91, 1.61) | 1.21 (0.91, 1.62) |
| Q3[1.40 (1.12-1.66)] | 1.44 (1.07, 1.95) * | 1.58 (1.12, 2.23) ** | 1.71 (1.20, 2.45) ** |  | 1.28 (0.98, 1.66) | 1.25 (0.94, 1.66) | 1.30 (0.97, 1.74) |
| Q4[2.11 (>1.66)] | 1.87 (1.40, 2.51) *** | 1.82 (1.28, 2.58) ** | 1.81 (1.25, 2.62) ** |  | 1.92 (1.49, 2.48) *** | 1.88 (1.42, 2.51) *** | 1.93 (1.43, 2.60) *** |
| *P* for trend | <0.001 | <0.01 | <0.01 |  | <0.001 | <0.001 | <0.001 |

Multifactor regression model was developed using group Q1 as the reference group. Calculate the median row trend test for each group.

OR: odds ratio, CI: confidence interval. ^*^: *P* <0.05, ^**^: *P* <0.01, ^***^: *P* <0.001.

**Table S6** Propensity score matching for outcome events with eGFR ≤60 mL/minute/1.73 m^2^

| **Features** | **eGFR>60** **mL/minute/1.73 m^2^(N=873)** | **eGFR≤60** **mL/minute/1.73 m^2^(N=873)** | ***P-*value** |
| --- | --- | --- | --- |
| Age | 68.00 (60.00, 77.00) | 70.00 (62.00, 80.00) | <0.001 |
| Gender |  |  | 0.002 |
| Men | 334 (38.26%) | 399 (45.70%) |  |
| Women | 539 (61.74%) | 474 (54.30%) |  |
| Diabetes |  |  | 0.401 |
| No | 553 (63.34%) | 536 (61.40%) |  |
| Yes | 320 (36.66%) | 337 (38.60%) |  |
| Smoking status |  |  | 0.188 |
| Never | 469 (53.72%) | 431 (49.37%) |  |
| Smoking former | 281 (32.19%) | 305 (34.94%) |  |
| Smoking now | 123 (14.09%) | 137 (15.69%) |  |
| Drinking status |  |  | 0.375 |
| Rarely drinker | 180 (20.62%) | 168 (19.24%) |  |
| Light drinker | 162 (18.56%) | 146 (16.72%) |  |
| Excessive drinker | 531 (60.82%) | 559 (64.03%) |  |
| Race/Ethnicity |  |  | <0.001 |
| Mexican American | 52 (5.96%) | 67 (7.67%) |  |
| Other Hispanic | 87 (9.97%) | 50 (5.73%) |  |
| Non-Hispanic White | 203 (23.25%) | 377 (43.18%) |  |
| Non-Hispanic Black | 396 (45.36%) | 305 (34.94%) |  |
| Other Race | 135 (15.46%) | 74 (8.48%) |  |
| Education |  |  | 0.739 |
| Less than high school | 265 (30.36%) | 258 (29.55%) |  |
| High school | 214 (24.51%) | 205 (23.48%) |  |
| More than high school | 394 (45.13%) | 410 (46.96%) |  |
| Marital status |  |  | 0.042 |
| Never married | 110 (12.60%) | 79 (9.05%) |  |
| Widowed/Divorced/  Separated | 368 (42.15%) | 367 (42.04%) |  |
| Married/Living  with partner | 395 (45.25%) | 427 (48.91%) |  |
| PLT | 212.00 (176.00, 250.00) | 215.00 (180.00, 258.00) | 0.094 |
| Hb | 13.10 (12.30, 13.90) | 13.30 (12.30, 14.30) | 0.011 |
| ALT | 19.00 (15.00, 25.00) | 19.00 (15.00, 25.00) | 0.390 |
| AST | 23.00 (19.00, 27.00) | 23.00 (20.00, 27.00) | 0.605 |
| TC | 4.84 (4.06, 5.61) | 4.71 (3.96, 5.51) | 0.029 |
| ALB | 42.00 (40.00, 44.00) | 42.00 (40.00, 44.00) | 0.595 |
| Glucose | 5.61 (5.05, 6.88) | 5.66 (5.05, 6.94) | 0.619 |
| HbA1c | 5.90 (5.50, 6.50) | 5.90 (5.60, 6.60) | 0.452 |
| SIRI | 0.55 (0.39, 0.85) | 1.25 (0.82, 1.87) | <0.001 |

All continuous numeric variables are expressed using medians (quartiles) and categorical variables are quantified as numbers (percentages).

PLT: platelets, ALT: alanine transaminase, AST: aspartate transaminase, TC: total cholesterol, ALB: Albumin, HbA1c: glycosylated hemoglobin, SIRI: Systemic Inflammation Response Index.

**Table S7** Propensity score matching for outcome events with ACR≥30 mg/g

| **Features** | **ACR<30** **mg/g(N=1075)** | **ACR≥30** **mg/g(N=1075)** | ***P-*value** |
| --- | --- | --- | --- |
| Age | 63.00 (54.00, 70.00) | 66.00 (55.00, 76.00) | <0.001 |
| Diabetes |  |  | 0.544 |
| No | 597 (55.53%) | 583 (54.23%) |  |
| Yes | 478 (44.47%) | 492 (45.77%) |  |
| Drinking status |  |  | 0.195 |
| Rarely drinker | 203 (18.88%) | 189 (17.58%) |  |
| Light drinker | 200 (18.60%) | 175 (16.28%) |  |
| Excessive drinker | 672 (62.51%) | 711 (66.14%) |  |
| Race/Ethnicity |  |  | <0.001 |
| Mexican American | 109 (10.14%) | 146 (13.58%) |  |
| Other Hispanic | 115 (10.70%) | 100 (9.30%) |  |
| Non-Hispanic White | 222 (20.65%) | 388 (36.09%) |  |
| Non-Hispanic Black | 488 (45.40%) | 309 (28.74%) |  |
| Other Race | 141 (13.12%) | 132 (12.28%) |  |
| Education |  |  | 0.557 |
| Less than high school | 352 (32.74%) | 350 (32.56%) |  |
| High school | 273 (25.40%) | 254 (23.63%) |  |
| More than high school | 450 (41.86%) | 471 (43.81%) |  |
| Marital status |  |  | 0.044 |
| Never married | 144 (13.40%) | 108 (10.05%) |  |
| Widowed/Divorced/  Separated | 377 (35.07%) | 406 (37.77%) |  |
| Married/Living  with partner | 554 (51.53%) | 561 (52.19%) |  |
| PLT | 220.00 (183.00, 259.00) | 220.00 (186.00, 267.00) | 0.127 |
| Hb | 13.40 (12.50, 14.30) | 13.60 (12.40, 14.70) | 0.006 |
| ALT | 21.00 (16.00, 28.00) | 20.00 (16.00, 27.00) | 0.264 |
| HDL-C | 1.32 (1.09, 1.60) | 1.27 (1.01, 1.55) | <0.001 |
| TG | 1.43 (0.93, 2.24) | 1.60 (1.05, 2.44) | <0.001 |
| TC | 4.94 (4.21, 5.69) | 4.81 (4.03, 5.66) | 0.029 |
| ALB | 42.00 (40.00, 44.00) | 42.00 (39.00, 44.00) | 0.095 |
| Glucose | 5.72 (5.05, 7.44) | 5.88 (5.16, 7.74) | 0.048 |
| HbA1c | 6.00 (5.60, 6.90) | 6.00 (5.60, 7.00) | 0.903 |
| SIRI | 0.53 (0.41, 0.71) | 1.30 (0.85, 1.99) | <0.001 |

All continuous numeric variables are expressed using medians (quartiles) and categorical variables are quantified as numbers (percentages).

PLT: platelets, ALT: alanine transaminase, HDL-C: high-density lipoprotein-cholesterol, TG: triglycerides, TC: total cholesterol, ALB: Albumin, HbA1c: glycosylated hemoglobin, SIRI: Systemic Inflammation Response Index.
